# French Medical Congress

**Published:** 1846-02

**Authors:** 


*French Medical Congress.*—An association of medical men, numbering now upwards of four thousand, has recently been formed in France, with a view of aiding and accelerating measures of medical reform, promised sometime since by the government. Early in November, the members of this association assembled in Paris, under the title of a 'Medical Congress,' and we are happy to learn that its meetings have been characterized by great zeal and intelligence, and have received all due countenance from the government itself—notwithstanding the jealousy with which it looks upon every popular combination having for its aim any reform of existing abuses, or rather interference with regular legislative action. M. Serres was President. Committees were appointed and reported on the topics of medical legislation, education and government; and resolutions have been adopted by the Congress, to be submitted to the Minister of Public Instruction, which embraces the views of that body on the entire subject of medical government.

The Committee appointed to examine the question of freedom of medical instruction, terminates with the following recommendations:

1st. Every member of the medical profession in France, should enjoy the right of teaching the medico-chirurgical sciences.

2d. In order that the freedom of medical teaching shall be as extended as possible, government should, in Paris and the principal towns of France, place a suitable locality, and all the material appliances necessary for practical teaching, at the disposal of every member of the medical profession.

3d. Private teaching should in no way infringe on the official education in the faculties and colleges, and should therefore confer no scholastic privilege, but merely aid in diffusing knowledge.

The first question discussed (relating to the exercise of the medical profession) was that of the existence of two orders of medical practitioners. The expediency of having an inferior grade of practitioners was denied, and the institution of officers of health was declared illegitimate and dangerous. This opinion was clearly announced in the following resolution, which was carried unanimously: That it is to be hoped, that in any future medical law one class of medical practitioners only will be recognized, that of doctors in medicine. That should such a decision be come to, existing officers of health might be allowed, during five years, to offer themselves as candidates for the M.D. degree at any of the faculties.

It was also decided at the same time, that the institution of district medical officers, to take care of the poor, was not necessary, and would be an attack on the liberty of the medical body. (This decision of the congress was arrived at in opposition to the wishes generally expressed in the documents received from the provinces.)—*Bulletin Med. Sci.*

